# Supplementary material for: Mid-Term Mortality in Older Anemic Patients with Type 2 Myocardial Infarction: Does Blood Transfusion sImprove Prognosis?
Source: J Clin Med. 2022 Apr 26;11(9):2423. doi: 10.3390/jcm11092423 (PMC9104580; doi:10.3390/jcm11092423)
Supplement: Supplementary file 1 [file jcm-11-02423-s001.zip › supplemental material.pdf]

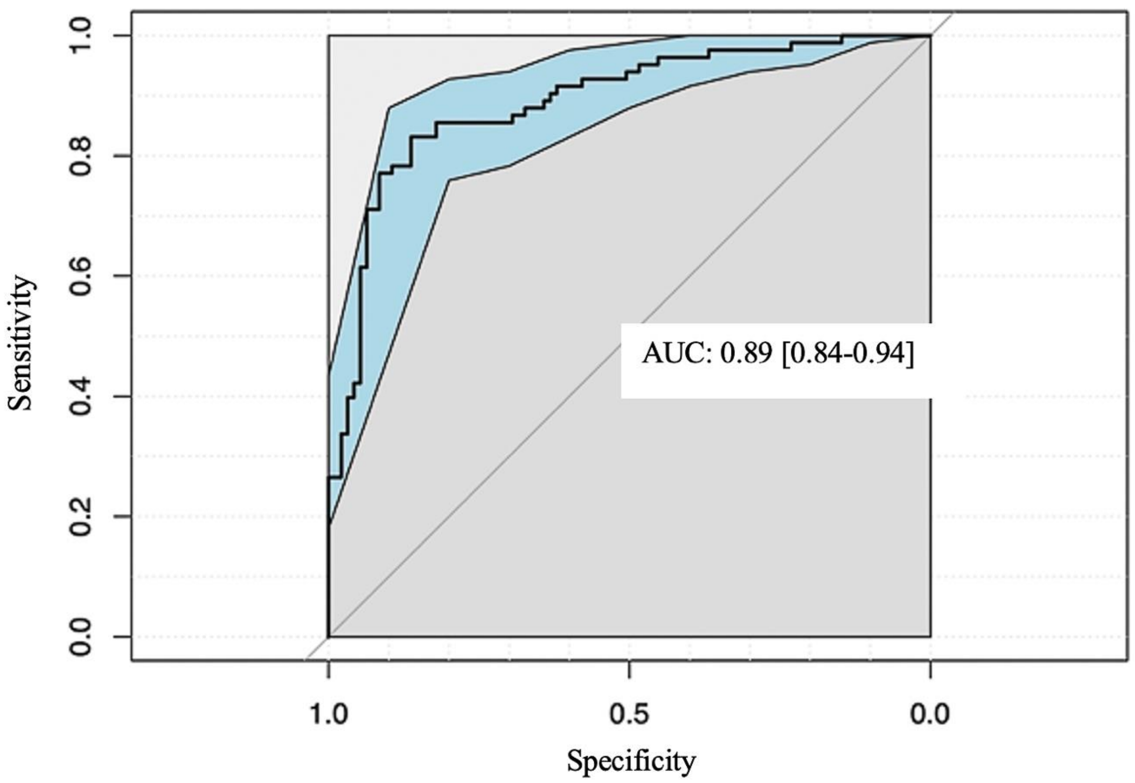

Figure S1. ROC curve analysis of the propensity score to predict red blood cell transfusion.

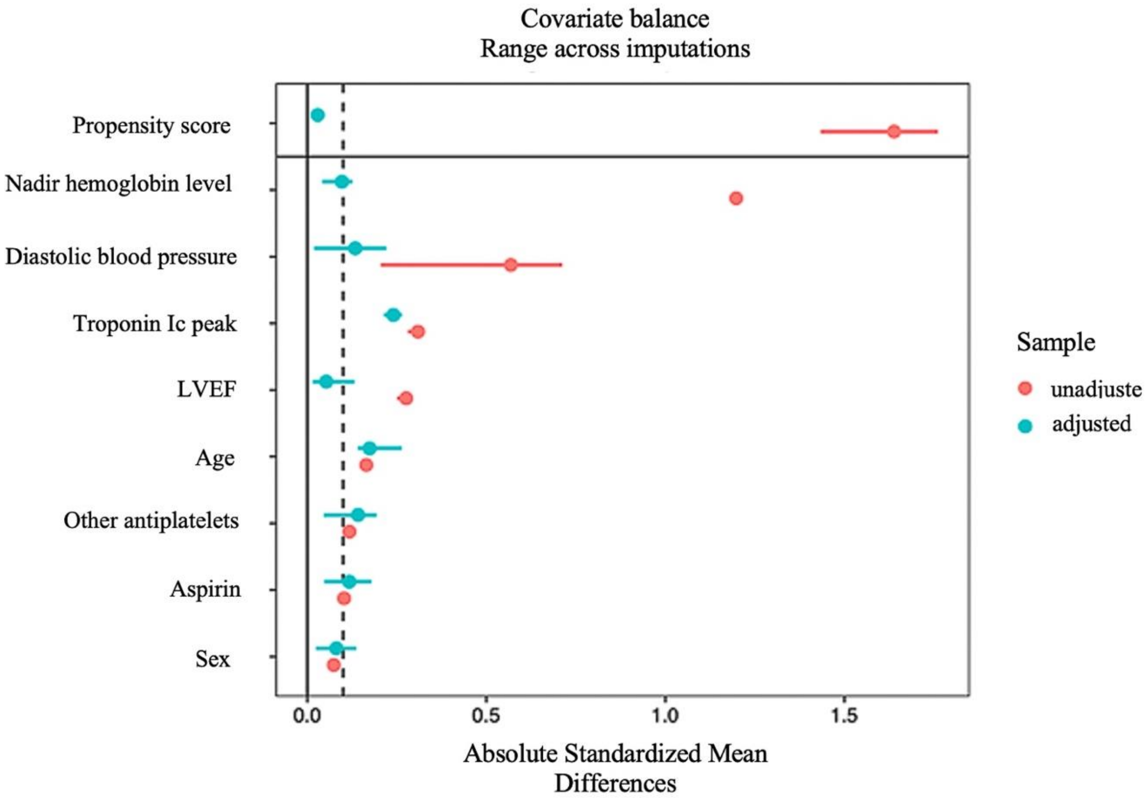

Figure S2. Covariate balance before and after weighting on propensity score.

Table S1. Difference of standard mean deviation (SMD) pre and post weighting.

Abbreviations: BMI, Body mass index; CAD, coronary artery disease; SBP, systolic blood pressure; DBP, diastolic blood pressure; CV, cardiovascular; LVEF, Left Ventricular Ejection Fraction; CKD-EPI, Chronic Kidney Disease Epidemiology Collaboration; NT -proBNP, N-terminal Pro-Brain Natriuretic Peptide.

	SMD pre-weighting	SMD Post-weighting
**Demographical data**		
Age (year)	0.184	0.173
Female	0.014	0.04
**CV risk factors**		
BMI (kg/m^2) (n=176)	0.219	0.14
Obesity (n=176)	0.257	0.04
Hypertension (n=178)	0.113	0.10
Diabetes (n=178)	0.026	0.136
Dyslipidemia (n=177)	0.025	0.087
Familial history of CAD (n=167)	0.133	0.007
Smoking (n=168)	0.072	0.09
**Medical history**		
Vascular history (n=178)	0.168	0.015
Myocardial infarction (n=178)	0.130	0.125
Coronary artery bypass graft (n=178)	0.016	0.052
Chronic kidney disease (n=174)	0.132	0.172
Thrombo-embolic event (n=176)	0.161	0.044
Atrial fibrillation (n=166)	0.011	0.042

Aortic stenosis (n=178)	0.364	0.1
Neurocognitive disorder (n=172)	0.072	0.009
Neoplasia (n=173)	0.024	0.181
**Chronic treatments**		
Aspirin (n=178)	0.098	0.057
Other antiplatelet (n=178)	0.147	0.058
Vitamin K inhibitor (n=178)	0.248	0.063
Oral anticoagulant (n=178)	0.153	0.012
Calcium inhibitor (n=178)	0.092	0.071
Angiotensin Receptor Blocker (n=178)	0.229	0.208
Angiotensin Converting Enzyme inhibitor (n=178)	0.144	0.002
**Clinical data on admission**		
Heart rate (b/min) (n=162)	0.105	0.34
SBP (mmHg) (n=163)	0.450	0.089
DBP (mmHg) (n=163)	0.643	0.286
Killip class > 1	0.074	0.085
LVEF (%) (n=177)	0.314	0.052
LVEF > 40% (n=177)	0.272	0.089
**Biological data**		
Hemoglobin at admission (g/dL) (n=178)	0.523	0.089
Nadir hemoglobin (g/dL) (n=178)	1.738	0.033
Drop in hemoglobin (n=178)	1.061	0.27
Creatinine (µmol/L) (n=176)	0.124	0.34

e-GFR (CKD-EPI) < 60 mL/min/1.73m^2 (n=176)	0.064	0.27
C reactive protein > 3 mg/L (n=175)	0.1	0.051
NT-proBNP (pg/mL) (n=169)	0.058	0.188
Troponin Ic peak (ng/mL) (n=175)	0.399	0.21
Coronary angiography (n=178)	0.384	0.033
